# Immunomodulatory potential of anti-idiotypic antibodies for the treatment of autoimmune diseases

**DOI:** 10.2144/fsoa-2020-0142

**Published:** 2020-10-29

**Authors:** Shing Yi Pan, Yvonne Cashinn Chia, Hui Rong Yee, Angelina Ying Fang Cheng, Clarice Evey Anjum, Yenny Kenisi, Mike KS Chan, Michelle BF Wong

**Affiliations:** 1 FCTI Biotech R&D GmbH Klosterstrasse 205, 67480 Edenkoben, Germany; 2 Baden R&D Laboratories GmbH Klosterstrasse 205, 67480 Edenkoben, Germany; 3 Baden Research & Testing Lab (Asia Pac) Sdn Bhd Unit 67 & 68, Block K, Alamesra Plaza Utama, 88450 Kota Kinabalu, Sabah, Malaysia

## Abstract

The immune system is a complex network of specialized cells and organs that recognises and reacts against foreign pathogens while remaining unresponsive to host tissues. This ability to self-tolerate is known as immunological tolerance. Autoimmune disease occurs when the immune system fails to differentiate between self and non-self antigens and releases autoantibodies to attack our own cells. Anti-idiotypic (anti-ID) antibodies are important in maintaining a balanced idiotypic regulatory network by neutralising and inhibiting the secretion of autoantibodies. Recently, anti-ID antibodies have been advanced as an alternative form of immunotherapy as they can specifically target autoantibodies, cause less toxicity and side effects, and could provide long-lasting immunity. This review article discusses the immunomodulatory potential of anti-ID antibodies for the treatment of autoimmune diseases.

## Lay abstract

The immune system protects the body against infections and diseases, such as by producing antibodies. Usually, these antibodies only attack pathogens, leaving healthy cells unharmed. However, autoimmune disease may develop when the immune system mistakenly recognises part of the body as foreign and produces antibodies to attack them. Antibodies that attack our own cells are called *autoantibodies*. Anti-idiotypic antibodies could be utilized to stop autoantibodies from attacking self cells with minimal side effects and long-lasting immunity. This review discusses anti-idiotypic antibodies usage as an alternative form of immunotherapy to inhibit autoantibodies in autoimmune disease.

The body’s immune system is a defence system that protects the host against pathogens or threats such as bacteria, parasites, fungi, viruses and the growth of tumour cells [1]. When foreign pathogens are detected, the immune system will produce proteins known as antibodies to attack them. In normal conditions, the immune system acts against non-self (foreign) antigens but does not act against our own (self) antigens. This lack of response against self-antigen induced by exposure of lymphocytes is known as immunological tolerance [2]. When immunological tolerance is disrupted, the body produces an abnormal response towards its own tissue, followed by the development of autoimmunity. Although the immune system can accurately recognise and eliminate invading pathogens in a healthy individual without causing severe harm towards the body, a different outcome is observed in immunocompromised individuals. The immune system of immunocompromised individuals often fails to distinguish between self and non-self antigen and begins to produce autoantibodies to attack host cells. This inappropriate response will eventually cause inflammation and tissue damage, which ultimately will lead to the development of different kinds of autoimmune diseases.

In general, autoimmune diseases are traditionally managed by the administration of immunosuppressing drugs such as corticosteroids, as well as mycophenolate mofetil (MMF), azathioprine (AZA) and cyclophosphamide [3]. The treatment strategy for autoimmune disease usually targets disease symptoms or depends on nonspecific immune suppression. However, long-term treatment with high doses of immunosuppressing drugs is often needed in the management of autoimmune disease, which then exposes the patient to life-threatening opportunistic infections and undesired side effects [4]. Therefore, attempts have been made to leverage on anti-idiotypic (anti-ID) antibodies for the management of autoimmune diseases over the past few years. It is well known that the presence of elevated circulating autoantibodies indicates dysregulation of the humoral immune response in patients with autoimmune disease. Autoantibodies are regulated not only by antigen but also by anti-ID antibodies that bind to the antigen-binding site of these autoantibodies. Indeed, anti-ID antibodies play an important role in maintaining a balanced idiotypic network in the human body. Anti-ID antibodies regulate both autoantibody binding and levels by neutralising autoantibodies and suppressing the production of autoantibodies, which eventually prevents the development of autoimmune disease. This hypothesis is supported by research that has shown that anti-ID antibodies are absent during periods of active autoimmune diseases (systemic lupus erythematosus [SLE] [5], rheumatoid arthritis [RA] [6] and Type 1 diabetes [T1D] [7]) and are only present in healthy individuals [8] or patients during the remission period (SLE [5], RA [6] and T1D [9]). An earlier review by Gorczynski and Hoffmann in 2017 discussed the application of anti-ID antibodies in preventive immunotherapy through the production of cytokines and the induction of regulatory T cells [10]. Thus far, the documented applications of anti-ID antibody in literature have focused on their applications in cancer treatments [11] and, in some cases, their potential as an alternative vaccine [10,12,13]. Hence, this review article will focus on discussing the function of anti-ID antibodies and their application in the treatment of autoimmune diseases.

## Autoimmune disease: a disruption in immunological tolerance

Autoimmune diseases have been indicated to be the 10th most common cause of mortality in developing countries. By definition, autoimmune diseases are characterised by malfunctioning of the body’s immune system, which causes the production of antibodies directed towards one’s own body tissues [14]. The hallmark of autoimmune diseases generally consists of the presence of self-reactive T cells, inflammation and production of autoantibodies [15]. Autoimmune disease happens when immune cells mistakenly acknowledge self-antigens (host tissue) as non-native molecules and thus develop specific antibodies known as autoantibodies. These autoantibodies have a strong affinity to self-antigens which ultimately disrupt the immunological tolerance of the body towards any foreign molecules [16].

Autoimmune diseases are generally categorised as either organ-specific or systemic, although cases of patients developing both forms of autoimmune diseases either sequentially or concurrently have been reported [17]. Organ-specific autoimmune diseases are defined as the misconduct of the normal immune responses towards self-antigen, resulting in overall consequential effects on the individual’s health status. It is characterised by the occurrence of lymphocytic infiltration and organ-specific autoantibodies, with the end result manifested either as endocrine hypofunction- or hyperfunction-related cases [18]. Examples of organ-specific autoimmune diseases include T1D, Graves’ disease and Addison’s disease. Most of the organ-specific autoimmune diseases are chronic, whereas others are deemed to be idiopathic. Autoantibodies in organ-specific autoimmune diseases are detectable in affected individuals years before the clinical manifestation of the disease [19]. Hence, the early screening of these antibodies will serve as an indicator of the impending risk within the family members of affected individuals.

By contrast, systemic autoimmune diseases target multiple organs, and chronic activation usually involves both the innate and adaptive immune cells [20]. Examples of systemic autoimmune diseases include SLE and RA. The diagnosis of systemic autoimmune disease proves to be a challenging task as the onset of the disease is nonspecific by nature (e.g., fever, malaise and arthromyalgias) [21]. Challenges in the diagnosis of systemic autoimmune disease can be seen in SLE, which has a complex heterogeneous clinical spectrum and the presence of a wide variety of autoantibodies [22]. It is therefore important to accurately detect the well-defined autoantibodies based on the serological profile of the affected individual.

Many studies have suggested that autoimmune diseases are highly dynamic due to their wide variability and different causative and pathogenic processes, causing most of the treatment strategies to fall short of expected complete remission. Currently, immunological tolerance is the main focus of many investigations to understand the mechanisms that maintain self-tolerance, prevent the development of autoimmune diseases and, most importantly, create strategies to induce tolerance in patients with autoimmune disease.

## Conventional treatments for autoimmune diseases & their limitations

Autoimmune diseases cannot be cured, but the presented symptoms can be alleviated and controlled with treatment. Patients diagnosed with autoimmune disease are traditionally managed through the administration of immunosuppressing drugs which can control the overactive immune response and relieve inflammation or pain. Although immunosuppressing drugs help to reduce symptoms, the overall impact on the disease is not effective, particularly in inducing remission and long-lasting cure [23–25]. Furthermore, long-term treatments with high doses are often required to keep the disease under control. Since the aim of these drugs is to reduce the immune response against the body’s own tissues, they eventually cause patients to become vulnerable to potentially life-threatening infections and increase their exposure to undesirable side effects associated with toxicities or the development of other conditions. For example, long-term use of corticosteroids has been reported to cause the development of osteoporosis and avascular necrosis [26]. In addition, long-term use of cyclophosphamide has been reported to cause severe side effects, including bone marrow suppression, impaired fertility and haemorrhagic cystitis, and increase the risk of developing cancer [27,28].

Besides the conventional use of immunosuppressing drugs, many physicians are currently inclined to treat patients using immunotherapy approaches, particularly in view of lower toxicities and increased specificity [3]. Several antigen-nonspecific therapies have gained considerable attention in the treatment of autoimmune disease, particularly those that target the common lymphocyte activation mechanism or the proinflammatory pathway. However, it is important to bear in mind that not every step of the proinflammatory immune pathway is necessarily a suitable therapeutic target, and only those that are rate limiting would likely be effective in ameliorating the progression of autoimmune diseases [29]. One such therapy that is known to be used in the treatment of RA targets TNF as the rate-limiting step, whereby TNFs are blocked with TNF-specific antibodies or soluble TNF receptors [30]. Although the blockade of TNF does reduce the production of many inflammatory mediators, it also potentially opens the flood gate to a multitude of other autoimmune diseases and infections, as well as increases the risk of developing lymphomas [31].

To address some of these issues and side effects associated with immunosuppressive drugs and most nonspecific immunotherapies, anti-ID antibodies could be used as an alternative form of immunotherapy to modulate the immune system. Although current literature discussing the use of anti-ID antibodies is largely dominated by their applications in cancer treatments [11] and, to a certain extent, their potential as alternative vaccines [32–34], their application and correlation with autoimmune diseases has also been widely reported (i.e., in SLE [35–38], RA [39,40] and T1D [41]) and will be discussed later in this review.

## Anti-ID antibodies in the idiotypic network theory

**Figure 1.**
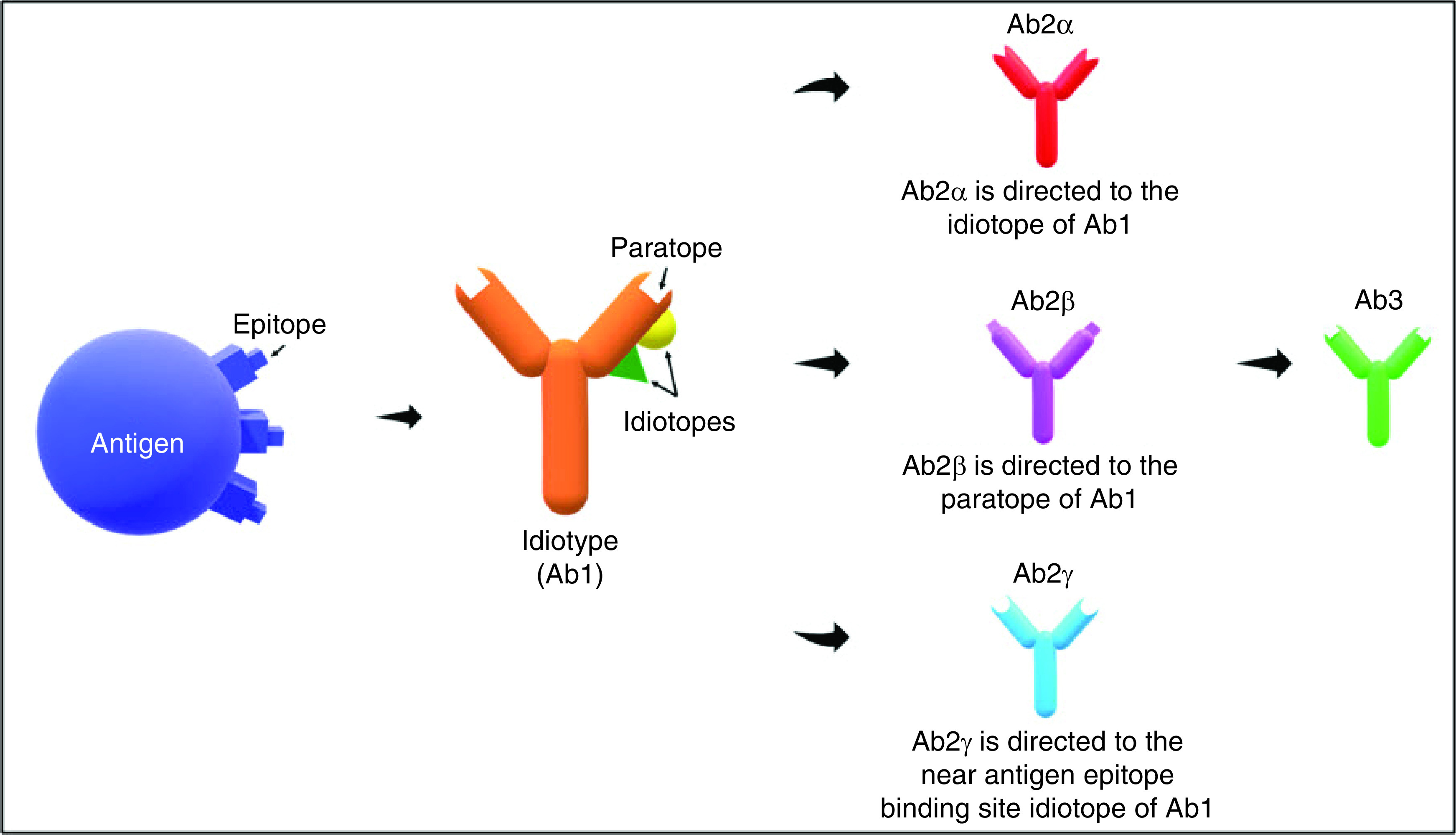
The idiotypic network theory. The presence of an antigenic determinant or epitope stimulate the production of Ab1 thus introduce the production of Ab2, which are classified as Ab2α, Ab2β or Ab2γ. Ab2α is directed to the idiotope of Ab1; Ab2β carry the internal image binding to antigen epitope and is directed to the paratope of Ab1; Ab2γ is directed to the near antigen epitope-binding site idiotope of Ab1.

The idiotypic network was formulated by Niels Jerne in 1974, comprising idiotypes (Ab1), anti-ID antibodies (also referred to as Ab2) and antigen-presenting cells [42,43]. According to the network theory, he postulated that the immune response might be regulated by the responses to idiotypes, which are unique determinants of the immunoglobulin or T-cell receptors (TCR) [43]. These idiotypes (Ab1) are able to identify antigens and are recognised by Ab2 to maintain homeostasis of the immune system [44]. During homeostasis, the presence of epitopes or antigenic determinants stimulate the production of antigen-specific antibodies (Ab1), which subsequently induce the production of anti-ID antibodies (Ab2) to maintain equilibrium [45]. Ab2 are classified into Ab2α, Ab2β or Ab2γ depending on their antigen-binding sites (Figure 1). Ab2α is directed to the idiotope of Ab1, whereas Ab2γ is directed to the near antigen epitope-binding site idiotope of Ab1. Additionally, Ab2β carry the internal image binding site to the paratope of Ab1 and bind to the complementarity determining region of Ab1, showing that Ab2β mimics structurally towards Ab1. Ab2 is able to stimulate the production of anti-anti-ID (Ab3) which have similar binding capacities as Ab1 [46]. This network theory allows the production of anti-ID antibodies to regulate autoantibodies by neutralising and inhibiting the secretion of autoantibodies, which eventually helps to prevent autoimmune diseases. Thus, many investigations focusing on Ab2 have been conducted to induce target-specific immune response [11,47–49]. Recently, two new categories of Ab2 were identified, known as Ab2δ and Ab2ε [50]. However, these two new categories of Ab2 are not discussed in this review due to the limited available information currently available.

The idiotypic network has been proven to have a fundamental role in the development of autoimmune diseases, whereby deficient idiotypic regulation of autoantibodies has been considered a major contributing factor for autoimmunity. It is well established that abnormalities in the idiotypic network could possibly result in the expression and expansion of autoantibodies. In the case of autoimmune diseases, the idiotypic network is disrupted and favours the expansion of Ab1 autoantibodies that respond to self-antigens [44]. Autoantibodies, maintained by both antigen and anti-ID antibodies that bind to the antigen-binding site of these autoantibodies, are usually found in low concentration in a healthy adult [8,51–53]. Hence, it is of general understanding that the presence of elevated circulating autoantibodies indicates dysregulation of the humoral immune response. Since anti-ID antibodies can act as the internal image of antigen epitopes, they can competitively bind to the autoantibodies in place of the antigen. Consequently, anti-ID antibodies help to neutralise and inhibit the production of autoantibodies, resulting in a balanced idiotypic network. Hence, maintenance of this equilibrium is essential to ensure that the immune system can effectively fight against exogenous antigen without attacking self-antigen that could potentially lead to the development of autoimmune diseases. In accordance with anti-ID antibodies serving an important role in immune homeostasis, approaches using anti-ID antibodies as vaccines for the treatment of autoimmune diseases are discussed in the following.

## Anti-ID antibodies for immunotherapy & their advantages

The unique property of the idiotypic network has paved the way for therapeutic treatments in many diseases, including autoimmune diseases. Comprehensive reviews of the use of anti-ID as cancer vaccines to induce humoral and/or cellular immune responses have been published [48,54]. Given that anti-ID antibodies mimic the shape of the antigen, researchers have been developing them as a new class of vaccine [10,12,13]. In 2017, Gorczynski and Hoffmann further discussed how anti-ID antibodies can potentially be used in preventive immunotherapy through the production of cytokines, induction of regulatory T cells, mitigation of graft intolerance, and attenuation of allergic and inflammatory bowel diseases [10].

Apart from the ability of anti-ID antibodies to inhibit antigen-antibody binding and suppress autoantibody production, the main advantage of anti-ID antibodies is their ability to selectively suppress only the complementary specific autoantibodies [55]. Due to the nonselective immunosuppressive nature of most conventional drugs and immunotherapies, both the protective and destructive immune responses are often suppressed, predisposing individuals to a higher risk of infectious diseases caused by opportunistic pathogens [4]. Thus, the use of anti-ID antibodies as immunotherapy could effectively suppress a specific autoimmune response while leaving the rest of the immune system functioning normally. Besides this, anti-ID antibodies induce a memory response (through the generation of T-helper memory cells) that not only persists following treatment but also prevents the occurrences of relapses [48] and promotes longer-lasting immunity [49]. In addition, since anti-ID antibodies naturally occur in the body [56] and the immune response elicited mimics those induced by nominal antigen [57], they are fairly safe and should not cause toxicity [58]. Vaccination with Ab2 has shown various advantages, as they bear the internal image of the antigen [59]. Furthermore, as anti-ID antibodies are obtained from the patient [60], incompatibility issues and risk of rejection by the recipient is practically nonexistent. Lastly, anti-ID antibodies have been reported to work well in those who do not respond well to conventional treatment in the case of autoimmune diseases. However, complications do arise in the development of anti-ID vaccines, as it is unidentified how long the anti-ID immunity could last [49].

## Anti-ID antibodies in SLE

SLE is a systemic autoimmune disease characterised by the uncontrolled production of multiple autoantibodies directed against nuclear antigens (DNA, nucleosomes and histones) and phospholipids. SLE affects many organs, including skin, kidneys, lungs, heart and other parts of the body. Although patients may be asymptomatic, the increase of these autoantibodies is indicative of imbalances in the idiotypic network. Many of these autoantibodies begin to develop before the clinical onset of SLE, and when patients are still asymptomatic.

For example, the titre of the anti-dsDNA antibody, considered to be the hallmark of SLE, has been detected as early as 2 years prior to any diagnosis in about half of the patients screened in a study involving 130 patients [61]. The titre of anti-dsDNA antibody usually continues to increase with the severity of SLE [62] and is often associated with ongoing inflammation and kidney damage [63]. However, the high titre of anti-dsDNA antibody can be neutralised and lowered through the binding of anti-ID antibodies which induces apoptosis of anti-dsDNA antibody-producing cells (e.g., G1–2 and M2–10) [36]. Once the anti-dsDNA antibody-producing cells have been successfully eliminated, the idiotypic network should regain equilibrium and return to normal.

An increase in the presence of ribosomal P phosphoprotein antibody (anti-P), another antibody that is highly specific to SLE [64], is also indicative of a problematic idiotypic network. As expected, anti-P antibodies have been found to be elevated during an active disease episode and have returned to a normal level during periods of remission [65]. SLE anti-ID antibodies are absent in most but not all SLE patients [5,56]; they can be found in healthy individuals, including relatives of SLE patients [8], those who were in contact with the patient [66] and other control individuals [8,66]. In healthy individuals, anti-P is present but masked by functional anti-ID; hence, it is said that these individuals are lacking overt anti-P but possess covert anti-P. According to Pan *et al.*, a functional idiotypic network for anti-P exists in SLE patients [67]. Still, dysfunctional anti-ID fails to mask the presence of overt anti-P, resulting in the loss of idiotypic regulation in SLE patients [67]. Alternatively, it is also likely that anti-IDs are absent in SLE patients, allowing overt anti-P to manifest itself and cause disruption in the idiotypic regulatory network of the affected individual [68]. In addition, the resurgence of autologous anti-ID antibody has been found in patients experiencing a remission from SLE [5], proving the importance of anti-ID antibodies in promoting patients’ spontaneous recovery from autoimmune diseases [69]. This possibly happens as the idiotypic network equilibrates itself due to the presence of functional anti-ID, permitting SLE patients to enter into remission.

The use of anti-ID antibodies as an SLE vaccine has been demonstrated in a small clinical trial following success in immunisation of a lupus mouse model, where it suppressed the development of nephritis, inhibited the production of anti-dsDNA antibodies and induced production of anti-ID antibodies [70]. Five out of the nine patients administered with the mouse anti-dsDNA monoclonal antibody were found to develop anti-ID antibodies within the first 3 months, without any adverse side effects and with patients remaining disease-free during the 2-year follow-up period [35]. This study demonstrates that idiotypic vaccination can successfully elicit a significant positive anti-ID response in SLE patients.

## Anti-ID antibodies in RA

In RA, the immune system produces antibodies that attack the lining of the joints, causing inflammation, pain and swelling. RA is the leading cause of joint inflammation with persisting pain and disability, resulting in a lifestyle bound by restricted movement and capabilities. Susceptibility to RA is contributed to by genetic factors (60%) [71], whereas the remaining 40% is from nongenetic risk factors including smoking habits, the diversity of gut microbiota, sedentary lifestyles and ethnic backgrounds [72].

The disease progression of RA begins with the formation of autoantibodies, namely rheumatoid factors (RFs) and anti-citrullinated protein antibody (ACPAs) directed towards self-antigens with increased levels of cytokines, chemokines and C-reactive protein in the blood circulation. Clinical experience has shown that both RFs and ACPAs are present in the sera samples of RA patients months or years before the advancement of the disease. The titres of these autoantibodies increased as the disease progressed, with most of the positive seroconversions occurring within 3 years of the start of symptoms [73]. Subsequently, this leads to synovial inflammation and the appearance of multiple clinical symptoms in RA patients [72]. Synovial inflammation is usually accompanied by the presence of hyperplasia of the joints, deformity caused by the cartilage and bone destruction, and systemic physiological changes including cardiovascular, pulmonary and skeletal disorders, as well as psychological effects [74]. However, the seroconversion of either RFs or ACPAs would still occur in some patients even after disease onset, usually within the first few years [75].

RFs are specifically directed towards the constant regions of IgG with different isotypes and affinities, and were first discovered about 70 years ago [76]. Furthermore, at least three RF isotypes, namely IgM, IgA and IgG, have been reported as being detectable in 52% of RA patients [40], making RFs a useful serological marker to be employed for the early diagnosis and management of RA. The use of anti-RF (anti-ID) antibodies from rabbits immunised with juvenile RA patients were found to bind specifically to IgM-RF, suggesting the anti-ID antibodies possess an internal image that can be recognised by RF with immunomodulatory properties [77]. Furthermore, anti-ID antibodies were developed against monoclonal RF (Ka m-RF), and results showed that patients with cross-reactive idiotypes treated with anti-ID showed marked reduction of RF *in vitro*, suggesting that the anti-ID antibodies may regulate RF production in RA patients [78]. These studies demonstrate that the development of anti-ID vaccines could be promising for the treatment of RA patients; however, further investigation should be carried out to further explore anti-IDs for the treatment of RA.

## Anti-ID antibodies in T1D

T1D, also known as insulin-dependent diabetes or juvenile diabetes, is a chronic disease characterised by little or no insulin produced by the pancreas. It is also an autoimmune disease in which the host’s immune system fails to recognise its own cells and attacks the insulin-producing cells in the pancreas. The autoimmune destruction of β cells will subsequently lead to insulin deficiency [41].

Autoantibodies against multiple β-cell antigens are the most significant predictor of the progression of T1D. The most well recognised autoantibody related to the development of T1D is GAD65Abs [79]. GAD65Abs can be detected a few years before the onset of diabetes and are found to be positive in more than 70% of patients at the time of diagnosis [80]. The development of autoantibodies usually happens before the onset of clinical manifestation of T1D, and indeed the rate of disease progression is directly proportional to autoantibody positivity [81].

However, GAD65Abs are also found to be present in sera of healthy individuals but masked by an epitope-specific anti-ID antibody [7]. In the healthy immune system, a balance between GAD65Abs, anti-ID antibodies and T cells is believed to contribute to the homeostasis of the adaptive immune response in the ‘network hypothesis’ [43], thereby protecting the host from GAD65-specific islet destruction. A temporary disturbance happens when there is a transient introduction of GAD65 into the anti-ID network. To maintain equilibrium, GAD65 will stimulate the secretion of GAD65Abs, which in turn stimulates the secretion of anti-ID antibodies from specific B cells [41,82]. Hence, these anti-ID antibodies will inhibit the binding capacity of GAD65Abs, preventing them from binding to GAD65. In healthy individuals, the circulating GAD65Abs in sera are not detectable using GAD65-specific methods, which may be due to the binding of anti-ID antibodies to the antigen-binding region of GAD65Abs. In contrast, the decline of anti-ID antibodies in T1D patients, causing the exposure of GAD65Abs, then can be served as critical serum markers in the diagnostics of diabetes [83].

A disturbance in the idiotypic regulatory network, either by a decrease in protective factors or elevated autoimmune elements, will lead to the development of autoimmune diseases. In T1D patients, the destruction of pancreatic β cells and the release of islet cell autoantigens contribute to the prolonged release of GAD65. GAD65 will continuously stimulate the secretion of GAD65Abs and, at the same time, trigger the production of anti-ID antibodies by B cells. However, the overproduction of GAD65 competes with anti-ID antibodies for binding to the GAD65 autoantibodies. When the autoantigen is present continuously, the GAD65Ab-specific B cells will receive a net stimulatory signal. In contrast, the anti-ID specific B cells will receive a net inhibitory signal, which then results in the decrease in anti-ID antibody secretion and, eventually, the apoptosis of anti-ID specific B cells. As a consequence, the loss of anti-ID antibodies will permanently eliminate the inhibition of GAD65 binding to GAD65Abs, which in turn allows the GAD65Ab-mediated antigen presentation and the continuous stimulation of autoreactive T cells [41,82].

Anti-ID antibodies have been proposed to be an important element in restoring the normal regulatory network in T1D patients by inhibiting GAD65Abs from binding to their antigen and potentially modulate T-cell response to GAD65. Thus, anti-ID antibodies can potentially function as an immune regulator in T1D. A study by Wang *et al.* in 2012 demonstrated that the administration of monoclonal anti-ID specific to GAD65 autoantibody in the non-obese diabetic mouse significantly lowered the severity of insulitis and consequently reduced the incidence rate of diabetes [84]. This finding supported the use of anti-ID antibodies as an idiotypic vaccination in performing the protective immune-modulatory role of GAD65Ab-specific anti-ID in the development of T1D.

## Challenges of anti-ID antibodies as vaccines

The development of anti-ID antibodies as vaccines poses many challenges. The process of choosing and developing the vaccines must be carefully performed, as they may demonstrate physiologic antagonistic effects [49]. Anti-ID antibodies developed by Gramsch *et al.* in 1988 were found to interact with the opiate receptors of the μ- and δ-types, and demonstrated opiate antagonistic activity [85]. Another challenge faced today for the development of vaccines is that it is unidentified how long the vaccine can sustain immunity [49]. To develop a better understanding of how these vaccines provoke antigen-specific immune response, more clinical trials must be carried out [49]. So far, the concept of anti-ID antibodies as vaccines has been demonstrated in several preclinical studies, mostly on cancer treatment. Active immunisation with anti-ID antibodies has been shown to induce significant antitumour activity in several animal disease models [86,87]. The efficacy of anti-ID as a vaccine against cancer has also been demonstrated in several clinical studies. For example, a phase 1b clinical trial using anti-ID antibodies was successfully conducted in 23 advanced colorectal cancer patients who had previously failed conventional therapy [11]. Another cancer vaccine, Racotumomab, was found to be safe and well tolerated in several clinical trials [48]. The toxicity of Racotumomab vaccine was then classified as grade 1, with a low rate of side effects observed in lung cancer patients [88].

Following clinical studies, these anti-ID antibodies as vaccines must obtain approval by regulatory bodies such as the US FDA and EMA. Although these vaccines have shown promising results, not many have received FDA approval for use in humans. For example, racotumomab (marketed as vaxira) was approved in Cuba and Argentina but is yet to be approved by the US FDA [54]. In 2010, sipuleucel-T became the first therapeutic cancer vaccine to receive FDA approval for the treatment of metastatic hormone-refractory prostate cancer [89]. In 2015, dinutuximab (marketed as unituxin) was granted orphan drug designation and approved by the FDA and EMA to be used as part of a first-line treatment on paediatric patients with high-risk neuroblastoma [90,91]. Subsequently, dinutuximab β (marketed as isquette), used as a second-line treatment of neuroblastoma, was also approved in 2017 [92].

In contrast, the application of anti-ID antibodies as vaccines in patients with autoimmune disease is still at an infant stage, although there has been some progress with some work entering the clinical trial stage. For example, as discussed earlier, SLE patients in a small clinical trial reported positive response upon vaccination with anti-ID antibodies and remained disease-free upon 2 years of follow-up [35]. Another study reported the success of two clinical trials using mouse anti-ID monoclonal antibody which successfully mimicked human melanoma antigen and elicited anti-tumour antibody in melanoma patients. Both of these clinical trials reported no toxicity nor allergic reactions, with one patient achieving complete remission [93].

Anti-ID antibodies also face production challenges. Anti-ID antibodies can be generated in the form of either monoclonal or polyclonal antibodies, each produced using different methods with their respective advantages and disadvantages. Generally, these antibodies do not rely on external antigen or fragments, which is the bottleneck factor in the production of conventional vaccines [49]. Monoclonal antibodies can be produced from hybridoma-based technologies which can recognise a single epitope of an antigen [94]. For example, in 1995, Foon *et al.* successfully generated a murine monoclonal anti-ID antibody which mimics a specific epitope on a tumour-associated antigen known as the carcinoembryogenic antigen using hybridoma-based technology [95]. However, monoclonal antibody production requires high technical skills and longer production time [96].

Meanwhile, polyclonal antibodies can be generated from immunised animals, which produce unique batch-specific biochemical and biophysical characteristics [54]. A polyclonal anti-ID antibody developed in goats against CO17-1A/GA733 was found to bind at two different epitopes in the colorectal cancer antigen as opposed to monoclonal antibodies that only bind to a single epitope of an antigen [97]. Although polyclonal antibodies are prone to batch-to-batch variability, their production times are relatively short compared with those of monoclonal antibodies [98]. Further research is also still required in terms of establishing a master cells bank for polyclonal anti-ID antibody production in a large culture tank [54]. Overall, the production of monoclonal anti-ID antibodies outweighs the few advantages of polyclonal anti-ID antibodies, especially in terms of their consistency and homogeneity.

Despite facing many challenges in the development of anti-ID antibodies as vaccines, research progress and clinical trials continue to produce promising results, suggesting that the scope of anti-ID vaccines could be extended beyond their traditional application in cancer treatment, particularly towards the management of autoimmune diseases.

## Conclusion

Autoantibody formation is the result of loss of self-tolerance and a part of the heightened immune response that leads to the development of autoimmune diseases. Understanding the mechanism of inflammation regulation will indeed bring significant clinical benefits for the diagnosis and treatment in autoimmune disease. The ideal treatment is to be able to target the fundamental cause of the disease, that is the loss of tolerance to self-antigen in autoimmune disease. The ability of anti-ID antibodies to neutralise and downregulate the secretion of pathologic autoantibodies has clinical implications in the prevention and treatment of autoimmune diseases. Therefore, anti-ID antibodies serve as promising vaccines in modulating the host response to autoantibodies in autoimmune diseases. The main advantage of anti-ID antibodies is that they target specifically to complementary autoantibodies, hence effectively suppressing a specific immune response while leaving the rest of the immune system functioning normally. Besides this, this immunotherapy should also result in less toxicity and fewer side effects compared with conventional therapies for autoimmune diseases. Most importantly, anti-ID antibodies could provide long-lasting immunity even in the absence of foreign antigen, which subsequently prevents the occurrence of relapses. However, the use of anti-ID antibody vaccines in autoimmune disease has yet to be well established, and more clinical trials must be carried out. This review article provides an overview of the function of anti-ID antibodies, presenting evidence that they could be used as an idiotypic vaccination, performing a protective immunomodulatory role. It also provides an overview of the challenges faced in this field.

## Future perspective

The days of nonspecific immunosuppressing drugs with side effects, high toxicity and limited efficacy could be numbered as the new era of immunotherapy pushes forward. One of the main goals for immunotherapy for autoimmune diseases is to restrict the immunosuppressive elements of the therapy so that they only act upon the specific autoimmune response without generating a state of generalised immunosuppression. Considering the immunomodulatory potential of anti-ID antibodies as antigen-specific immunotherapy as referred to in this review article, they are likely to play a key role in future attempts to generate an effective treatment for autoimmune diseases.

**Executive summary**

- Immunological tolerance is a state of unresponsiveness of the immune system to antigens that have the capacity to cause a specific immune response in the human body.
- When immunological tolerance is disrupted, the immune system fails to distinguish between self and non-self antigens and develop autoantibodies to attack host cells, which eventually results in the development of autoimmune disease.
- Traditional treatments for autoimmune disease have relied on immunosuppressing drugs that suppress patient immune responses. However, long-term use of these drugs often brings undesirable side effects associated with toxicities and predisposes the patient to life-threatening opportunistic infections. Besides this, the overall impact of these drugs on autoimmune disease is not effective, especially in inducing remission and long-lasting immunity.
- Anti-idiotypic (anti-ID) antibodies and autoantibodies interact with each other to maintain a balanced idiotypic regulatory network. Anti-ID antibodies can competitively bind to the autoantibodies in place of the antigen. Hence, they help to neutralise and inhibit the release of autoantibodies for the maintenance of tolerance.
- Over the past few years, anti-ID antibodies have been used as a vaccine in the context of cancer. The capability of anti-ID antibodies in conferring antigen-specific immune tolerance with no compromise in the ability to elicit immune responses to other antigens has paved the way for many therapeutic processes in autoimmune diseases.
